# Supplementary material for: Break down the decentralization-security-privacy trilemma in management of distributed energy systems
Source: Nat Commun. 2024 May 27;15:4508. doi: 10.1038/s41467-024-48860-7 (PMC11130155; doi:10.1038/s41467-024-48860-7)
Supplement: Supplementary file 1 — Supplementary Information [file 41467_2024_48860_MOESM1_ESM.pdf]

# Supplementary Information: Break down the Decentralization-Security-Privacy Trilemma in Management of Distributed Energy Systems

Qinghan Sun[1], Huan Ma[1], Tian Zhao[1,2], Yonglin Xin[1] and Qun Chen[1,2*]

[1*]Key Laboratory for Thermal Science and Power Engineering of Ministry of Education, Department of Engineering Mechanics, Tsinghua University, Beijing, 100084, China.
[2]School of Energy Storage Science and Engineering, North China University of Technology, Beijing, 100144, China.

*Corresponding author(s). E-mail(s): chenqun@tsinghua.edu.cn;
Contributing authors: sqh20@mails.tsinghua.edu.cn;
mahuan@mail.tsinghua.edu.cn; zhaotianfred@gmail.com;
xyl18@mails.tsinghua.edu.cn;

# Supplementary Note 1: Subproblem formulation

The problem of optimal dispatch of power grid with various generation equipments has been studied by a lot of researchers[1,2]. In this work, we consider the general structure of DESs connected with power grid shown in Supplementary Fig. 1.

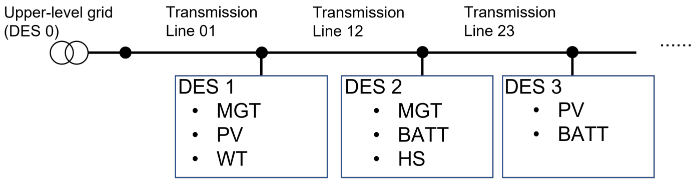


**Supplementary Fig. 1** The general structure of connected DESs. The upper-level grid is also treated as a special DES. Each DES may be equipped with micro-gas-turbines(MGT), photovoltaic panels(PV), wind turbines(WT), batteries(BATT), heat storage devices(HS), or any other distributed energy sources.

The power grid is assumed to be a radial distribution network. Without loss of generality, we assume that each node(bus) of the network connects a unique DES, and no longer distinguish between the terms node and DES. The upper-level grid connected at some node is treated as a special DES with number 0.

According to the linearized distflow model[3,4], the constraints describing the power transmission are:

$$\sum_{j:j=pa(i)} P_{ji,t} = \sum_{k:i=pa(k)} (P_{ik,t} + l_{ik,t}R_{ik}) + P_{i,t} \tag{1}$$

$$\sum_{j:j=pa(i)} Q_{ji,t} = \sum_{k:i=pa(k)} (Q_{ik,t} + l_{ik,t}X_{ik}) + Q_{i,t} \tag{2}$$

$$v_{i,t} = v_{j,t} - 2\left(R_{ji}P_{ji,t} + X_{ji}Q_{ji,t}\right) \tag{3}$$

where $t = 1, 2, ..., T$ is the time-related subscript and $T$ is the total number of discrete operation decisions in a day. $j = pa(i)$ indicates that node $j$ is the parent of $i$ in the tree-like grid. Herein, the term "parent" of node $i$ refers to the first DES node on the route from DES $i$ to the upper-level grid. For example, in Supplementary Fig. 1, DES 1 is the parent of DES 2, and DES 2 is the parent of DES 3. $P_{ji,t}$ and $Q_{ji,t}$ denote active and reactive power flow at time section $t$, while $P_{i,t}$ and $Q_{i,t}$ represent the net active and reactive power extracted from the power grid by DES $i$ at $t$. $v_{i,t}$ and $l_{i,t}$ are the squared magnitudes of nodal voltage and transmission line current. $R_{ij}$ and $X_{ij}$ represent the impedances of lines. The safety constraints are:

$$\underline{v_i} \le v_{i,t} \le \overline{v_i} \tag{4}$$

The DESs are prosumers of power and heat. Their power and heat balance equations are listed as follows:

$$P_{i,t} = P_{i,t}^{l} + P_{i,t}^{\mathrm{HP}} - P_{i,t}^{\mathrm{MGT}} - P_{i,t}^{\mathrm{WT}} - P_{i,t}^{\mathrm{PV}} + P_{i,t}^{\mathrm{BATT\text{-}s}} - P_{i,t}^{\mathrm{BATT\text{-}r}} \tag{5}$$

$$Q_{i,t} = Q_{i,t}^{l} - Q_{i,t}^{\mathrm{MGT}} - Q_{i,t}^{\mathrm{WT}} - Q_{i,t}^{\mathrm{PV}} \tag{6}$$

$$H_{i,t}^{l} = H_{i,t}^{\mathrm{MGT}} + H_{i,t}^{\mathrm{HP}} + H_{i,t}^{\mathrm{HS,r}} - H_{i,t}^{\mathrm{HS,s}} \tag{7}$$

where $P$, $Q$ and $H$ represent active power, reactive power and heat respectively. The superscript $l$ denotes loads. The other terms with superscripts such as MGT represent the generation of corresponding devices in DES $i$. MGT represents distributed micro gas-fired turbines for combined heat and power generation. HP represents heat pump. WT stands for wind turbine. PV means photovoltaic panels. BATT and HS respectively represent batteries and heat storage devices. -r means discharging or heat release and -s stands for Battery charging or heat storage. The operational constraints are:

$$\underline{P}_i^{\mathrm{dev}} \le P_{i,t}^{\mathrm{dev}} \le \overline{P}_i^{\mathrm{dev}}, \mathrm{dev} = \text{MGT, HP, WT, PV, BATT-r or BATT-s} \tag{8}$$

$$Q_{i,t}^{\mathrm{dev}} = P_{i,t}^{\mathrm{dev}} \tan \varphi_i^{\mathrm{dev}}, \mathrm{dev} = \text{MGT, WT or PV} \tag{9}$$

$$\underline{H}_i^{\mathrm{dev}} \le H_{i,t}^{\mathrm{dev}} \le \overline{H}_i^{\mathrm{dev}}, \mathrm{dev} = \text{HS-s, HS-r} \tag{10}$$

$$H_{i,t}^{\mathrm{MGT}} = k_i^{\mathrm{MGT}} P_{i,t}^{\mathrm{MGT}} \tag{11}$$

$$H_{i,t}^{\mathrm{HP}} = \mathrm{COP}_i^{\mathrm{HP}} P_{i,t}^{\mathrm{HP}} \tag{12}$$

$$\mathrm{SOC}_{i,t}^{\mathrm{BATT}} = \mathrm{SOC}_{i,t-1}^{\mathrm{BATT}} + P_{i,t}^{\mathrm{BATT\text{-}s}} \eta_s - P_{i,t}^{\mathrm{BATT\text{-}r}} / \eta_r \tag{13}$$

$$0 \le \mathrm{SOC}_{i,t}^{\mathrm{BATT}} \le \overline{\mathrm{SOC}_i^{\mathrm{BATT}}} \tag{14}$$

$$\mathrm{SOH}_{i,t}^{\mathrm{HS}} = \left(\mathrm{SOH}_{i,t-1}^{\mathrm{HS}} + H_{i,t}^{\mathrm{HS\text{-}s}} - H_{i,t}^{\mathrm{HS\text{-}r}}\right)(1 - \mathrm{loss}_i \Delta t) \tag{15}$$

$$0 \le \mathrm{SOH}_{i,t}^{\mathrm{HS}} \le \overline{\mathrm{SOH}_i^{\mathrm{HS}}} \tag{16}$$

where $\varphi$ represent the power angles. $k_i^{\mathrm{MGT}}$ is the heat-to-power generation ratio of MGT $i$ and $\mathrm{COP}_i^{\mathrm{HP}}$ denotes the coefficient of performance of heat pump $i$. $k_i^{\mathrm{MGT}}$ and $\mathrm{COP}_i^{\mathrm{HP}}$ are considered constant. SOC stands for state of charge of batteries and $\eta$ is the efficiency of charging or discharging. $\mathrm{SOH}_{i,t}^{\mathrm{sto}}$ is the state of heat storage devices and $\mathrm{loss}_i$ is the coefficient representing heat loss. $\Delta t$ is the time interval of dispatch and takes 1 hour.

As for the generation cost, we consider those of the gas-fired MGT and the renewables:

$$C_i^{\mathrm{DES}} = \sum_{t=1}^{T} \left[ f_i^{\mathrm{MGT}}\left(P_{i,t}^{\mathrm{MGT}}\right) + f_i^{\mathrm{WT}}\left(P_{i,t}^{\mathrm{WT}}\right) + f_i^{\mathrm{PV}}\left(P_{i,t}^{\mathrm{PV}}\right) \right] \tag{17}$$

where $f_i^{\mathrm{MGT}}(\cdot)$, $f_i^{\mathrm{WT}}(\cdot)$ and $f_i^{\mathrm{PV}}(\cdot)$ are quadratic cost functions. Since $H_{i,t}^{\mathrm{MGT}}$ is coupled with $P_{i,t}^{\mathrm{MGT}}$ through the heat-to-power ratio, $f_i^{\mathrm{MGT}}(\cdot)$ is treated as

a univariate function of $P_{i,t}^{\mathrm{MGT}}$. Besides, the coefficients of $f_i^{\mathrm{WT}}(\cdot)$ and $f_i^{\mathrm{PV}}(\cdot)$ are relatively small compared to those of $f_i^{\mathrm{MGT}}(\cdot)$ considering they represent the cost of renewable sources.

All of the decision variables with subscripts $i$ and $j$ simultaneously, such as $P_{ji,t}$ and $Q_{ij,t}$, are duplicated and included in both $\mathbf{x}_{c,i,j}$ and $\mathbf{x}_{c,j,i}$, which we define as the coupling variables between $i$ and $j$. Besides, the decision variables related to nodal voltage, i.e., $v_{i,t}$ are also duplicated and involved in both $\mathbf{x}_{c,pa(i),i}$ and $\mathbf{x}_{c,i,pa(i)}$. According to the definition above, one important constraint describing consensus between DES agents is,

$$\mathbf{x}_{c,i,j} - \mathbf{x}_{c,j,i} = 0, \quad \forall i \text{ and } j \in \mathcal{N}_i \tag{18}$$

where $\mathcal{N}_i$ denotes the set of neighbouring DESs of DES $i$.

The other decision variables uniquely labeled by $i$, such as $P_{i,t}$, $Q_{i,t}$, $P_{i,t}^{\mathrm{dev}}$, $\mathrm{SOC}_{i,t}^{\mathrm{BATT}}$, and $\mathrm{SOH}_{i,t}^{\mathrm{HS}}$ are denoted as $\mathbf{x}_{d,i}$ in the main text of the paper.

To conclude, the overall cost minimization problem of multiple connected DESs is shown as follows:

$$\min_{\substack{\mathbf{x}_i=[\mathbf{x}_{d,i},\mathbf{x}_{c,i,j},\forall j\in\mathcal{N}_i],\forall i\\ \mathbf{e}_{c,i,j},\forall i \text{ and } j\in\mathcal{N}_i}} \sum_{\text{DES } i} \left(\frac{1}{2}\mathbf{x}_{d,i}^{\top}\mathbf{H}_{d,i}\mathbf{x}_{d,i} + \mathbf{c}_{d,i}^{\top}\mathbf{x}_{d,i}\right) \tag{19a}$$

$$s.t. \quad \mathbf{A}_{d,i}\mathbf{x}_{d,i} + \sum_{j\in\mathcal{N}_i} \mathbf{A}_{c,i,j}\mathbf{x}_{c,i,j} = \mathbf{b}_i, \quad \forall i \tag{19b}$$

$$\mathbf{G}_{d,i}\mathbf{x}_{d,i} + \sum_{j\in\mathcal{N}_i} \mathbf{G}_{c,i,j}\mathbf{x}_{c,i,j} \leq_{\mathcal{K}_i} \mathbf{h}_i, \quad \forall i \tag{19c}$$

$$\mathbf{x}_{c,i,j} - \mathbf{e}_{c,i,j} = 0, \quad \forall i \text{ and } j \in \mathcal{N}_i, \quad \forall i \tag{19d}$$

$$\mathbf{e}_{c,i,j} - \mathbf{e}_{c,j,i} = 0, \quad \forall i \text{ and } j \in \mathcal{N}_i, \quad \forall i \tag{19e}$$

where $i$ and $j$ mark the index of involved DESs. (22c) and (22d) are equality and inequality constraints compacting (1) to (16). The quadratic cost functions $f_i^{\mathrm{MGT}}(\cdot)$, $f_i^{\mathrm{WT}}(\cdot)$ and $f_i^{\mathrm{PV}}(\cdot)$ are reorganized into the matrix $\mathbf{H}_{d,i}$ and $\mathbf{c}_{d,i}$. $\mathbf{e}_{c,i,j}$ represents the newly introduced auxiliary variables.

In Alternating Direction of Multipliers(ADMM)[5–7], the above problem (19) can be calculated as follows:

S1 : Relax constraint (19d) to obtain the following augmented lagrangian problem,

$$\min_{\substack{\mathbf{x}_i=[\mathbf{x}_{d,i},\mathbf{x}_{c,i,j},\forall j\in\mathcal{N}_i],\forall i\\ \mathbf{e}_{c,i,j},\forall i \text{ and } j\in\mathcal{N}_i}} \sum_{\text{DES } i} \left(\frac{1}{2}\mathbf{x}_{d,i}^{\top}\mathbf{H}_{d,i}\mathbf{x}_{d,i} + \mathbf{c}_{d,i}^{\top}\mathbf{x}_{d,i}\right) + \tag{20a}$$

$$\sum_{i,j\in\mathcal{N}_i} \left[(\boldsymbol{\lambda}_{i,j}^{\top}(\mathbf{x}_{c,i,j} - \mathbf{e}_{c,i,j}) + \frac{1}{2}\|\mathbf{x}_{c,i,j} - \mathbf{e}_{c,i,j}\|_{\boldsymbol{\Theta}}^2\right] \tag{20b}$$

$$s.t.\quad \mathbf{A}_{d,i}\mathbf{x}_{d,i} + \sum_{j\in\mathcal{N}_i} \mathbf{A}_{c,i,j}\mathbf{x}_{c,i,j} = \mathbf{b}_i, \quad \forall i \tag{20c}$$

$$\mathbf{G}_{d,i}\mathbf{x}_{d,i} + \sum_{j\in\mathcal{N}_i} \mathbf{G}_{c,i,j}\mathbf{x}_{c,i,j} \leq_{\mathcal{K}_i} \mathbf{h}_i, \quad \forall i \tag{20d}$$

$$\mathbf{e}_{c,i,j} - \mathbf{e}_{c,j,i} = 0, \quad \forall i \text{ and } j \in \mathcal{N}_i, \quad \forall i \tag{20e}$$

where $\boldsymbol{\lambda}_{i,j}$ represent lagrangian multipliers. $\|\cdot\|^2_{\boldsymbol{\Theta}}$ stands for the quadratic form defined by positive-definite diagonal matrix $\boldsymbol{\Theta}$.

S2 : Solve $\mathbf{x}_i = [\mathbf{x}_{d,i}, \mathbf{x}_{c,i,j}, \forall j \in \mathcal{N}_i], \forall i$ which minimizes (20) when fixing $\mathbf{e}_{c,i,j}$ and $\boldsymbol{\lambda}_{i,j}$

S3 : Solve $\mathbf{e}_{c,i,j}$ for $\forall i$ and $j \in \mathcal{N}_i$ which minimizes (20) when fixing $x_i$ and $\boldsymbol{\lambda}_{i,j}$

S4 : Update $\boldsymbol{\lambda}_{i,j}$ with dual ascent,

$$\boldsymbol{\lambda}_{i,j} := \boldsymbol{\lambda}_{i,j} + \boldsymbol{\Theta}\left(\mathbf{x}_{c,i,j} - \mathbf{e}_{c,i,j}\right) \tag{21}$$

S5 : Return to S2 until convergence of $x_i$, $\mathbf{e}_{c,i,j}$ and $\boldsymbol{\lambda}_{i,j}$.

Considering the structure of (20) after introducing auxiliary $\mathbf{e}_{c,i,j}$, S2-S4 can be implemented in a fully decentralized manner. Specifically, in S2, all of the edge devices owned by the DESs solve the following problem for $\mathbf{x}_i$,

$$\min_{\mathbf{x}_i=[\mathbf{x}_{d,i},\mathbf{x}_{c,i,j}\forall j\in\mathcal{N}_i]} \left(\frac{1}{2}\mathbf{x}_{d,i}^\top \mathbf{H}_{d,i}\mathbf{x}_{d,i} + \mathbf{c}_{d,i}^\top \mathbf{x}_{d,i}\right) + \tag{22a}$$

$$\sum_{j\in\mathcal{N}_i}\left[(\boldsymbol{\lambda}_{i,j}^\top(\mathbf{x}_{c,i,j} - \mathbf{e}_{c,i,j}) + \frac{1}{2}\|\mathbf{x}_{c,i,j} - \mathbf{e}_{c,i,j}\|^2_{\boldsymbol{\Theta}}\right] \tag{22b}$$

$$s.t.\quad \mathbf{A}_{d,i}\mathbf{x}_{d,i} + \sum_{j\in\mathcal{N}_i} \mathbf{A}_{c,i,j}\mathbf{x}_{c,i,j} = \mathbf{b}_i \tag{22c}$$

$$\mathbf{G}_{d,i}\mathbf{x}_{d,i} + \sum_{j\in\mathcal{N}_i} \mathbf{G}_{c,i,j}\mathbf{x}_{c,i,j} \leq_{\mathcal{K}_i} \mathbf{h}_i \tag{22d}$$

which is exactly what we have presented in the Methods section of the paper.

In S3, the solution to (20) simply yields $\mathbf{e}_{c,i,j}^{\text{opt}} = (\mathbf{x}_{c,i,j} + \boldsymbol{\Theta}^{-1}\boldsymbol{\lambda}_{i,j} + \mathbf{x}_{c,j,i} + \boldsymbol{\Theta}^{-1}\boldsymbol{\lambda}_{j,i})/2$ and can be calculated by DES $i$ and $j$ respectively.

In our work, the subproblems (22) are first formulated and encrypted by dedicated trusted edge devices. Afterwards, they are broadcast to blockchain and computation parties and solved through interior point methods. Finally, the encrypted optimal value of $\mathbf{x}_i, \forall i$ is returned to DES owners, where each edge device $i$ updates $\mathbf{e}_{c,i,j}$ and $\boldsymbol{\lambda}_{i,j}$ for $\forall j \in \mathcal{N}_i$ in a fully decentralized manner.

In practical implementations, all of the primal variables $\mathbf{x}_i$, $\mathbf{e}_{c,i,j}$ are initialized to 0 vectors. The dual variables $\boldsymbol{\lambda}_{i,j}$ related to power transmission can be initialized to the Time-of-Use price table. As a result, the update of $\mathbf{e}_{c,i,j}$ can be further simplified to $\mathbf{e}_{c,i,j}^{\text{opt}} = (\mathbf{x}_{c,i,j} + \mathbf{x}_{c,j,i})/2$ and $\boldsymbol{\lambda}_{i,j} + \boldsymbol{\lambda}_{j,i} = 0$ always holds in the iteration.

# Supplementary Note 2: Proof of the equivalence between the original problem and the obfuscated problem

We rewrite (22) into (23) and prove the following proposition: The following two optimization problems (23) and (24) (i.e. problem (3) and (6) in the Methods section) have the same optimum considering the relationship (25)(labelled as (7) in the main text). and the optimizer of (23) can be recovered from that of (24).

$$\min \quad \frac{1}{2}\mathbf{x}_i^\top \mathbf{H}_i \mathbf{x}_i + \mathbf{c}_i^\top \mathbf{x}_i + a_i \tag{23a}$$

$$s.t. \quad \mathbf{A}_i \mathbf{x}_i = \mathbf{b}_i \tag{23b}$$

$$\mathbf{G}_i \mathbf{x}_i \leq_{\mathcal{K}_i} \mathbf{h}_i \tag{23c}$$

$$\min \quad \frac{1}{2}\mathbf{y}_i^\top \mathbf{H}'_i \mathbf{y}_i + \mathbf{c}'^\top_i \mathbf{y}_i + a'_i \tag{24a}$$

$$s.t. \quad \mathbf{G}'_i \mathbf{y}_i \leq_{\mathcal{K}_i} \mathbf{h}'_i \tag{24b}$$

$$\mathbf{H}'_i = \mathbf{R}_i^\top \mathbf{N}_i^\top \mathbf{H}_i \mathbf{N}_i \mathbf{R}_i \tag{25a}$$

$$\mathbf{c}'_i = \left(\mathbf{c}_i + \mathbf{x}_i^{0\top} \mathbf{H}_i\right) \mathbf{N}_i \mathbf{R}_i \tag{25b}$$

$$a'_i = \frac{1}{2}\mathbf{x}_i^{0\top} \mathbf{H}_i \mathbf{x}_i^0 + \mathbf{c}_i^\top \mathbf{x}_i^0 \tag{25c}$$

$$\mathbf{G}'_i = \mathbf{G}_i \mathbf{N}_i \mathbf{R}_i \tag{25d}$$

$$\mathbf{h}'_i = \mathbf{h}_i - \mathbf{G}_i \mathbf{x}_i^0 \tag{25e}$$

*Proof* Let $\mathbf{y}_i^*$ and $s_i^*$ be the optimizer and optimum of (24). In other words, we have $\mathbf{G}'_i \mathbf{y}_i^* \leq_{\mathcal{K}_i} \mathbf{h}'_i$ and $s_i^* = \frac{1}{2}\mathbf{y}_i^{*\top} \mathbf{H}'_i \mathbf{y}_i^* + \mathbf{c}'^\top_i \mathbf{y}_i^* + a'_i$. Besides, for $\forall \mathbf{y}_i$ s.t. $\mathbf{G}'_i \mathbf{y}_i \leq_{\mathcal{K}_i} \mathbf{h}'_i$, $s_i^* \leq \frac{1}{2}\mathbf{y}_i^\top \mathbf{H}'_i \mathbf{y}_i + \mathbf{c}'^\top_i \mathbf{y}_i + a'_i$. Let $\mathbf{x}_i^*$ be defined as $\mathbf{x}_i^* = \mathbf{N}_i \mathbf{R}_i \mathbf{y}_i^* + \mathbf{x}_i^0$.

(Feasibility of $\mathbf{x}_i^*$) Recall that $\mathbf{N}_i \in \mathbb{R}^{n \times (n-m)}$ has full column rank and defines the null space of $\mathbf{A}_i$, and that $\mathbf{A}_i \mathbf{x}_i^0 = \mathbf{b}_i$. Then it is obvious that $\mathbf{A}_i \mathbf{x}_i^* = \mathbf{A}_i(\mathbf{N}_i \mathbf{R}_i \mathbf{y}_i^* + \mathbf{x}_i^0) = 0 + \mathbf{A}_i \mathbf{x}_i^0 = \mathbf{b}_i$ and that $\mathbf{G}_i \mathbf{x}_i^* = \mathbf{G}_i(\mathbf{N}_i \mathbf{R}_i \mathbf{y}_i^* + \mathbf{x}_i^0) = \mathbf{G}'_i \mathbf{y}_i^* + \mathbf{G}_i \mathbf{x}_i^0 \leq_{\mathcal{K}_i} \mathbf{h}_i$. Therefore, $\mathbf{x}_i^*$ is a feasible solution of (23).

(Optimality of $\mathbf{x}_i^*$) Consider $\forall \tilde{\mathbf{x}}_i$ s.t. $\mathbf{A}_i \tilde{\mathbf{x}}_i = \mathbf{b}_i$ and $\mathbf{G}_i \tilde{\mathbf{x}}_i \leq_{\mathcal{K}_i} \mathbf{h}_i$. $\mathbf{A}_i \tilde{\mathbf{x}}_i = \mathbf{b}_i$ indicates that $\tilde{\mathbf{x}}_i$ is a solution of the non-homogeneous linear system $\mathbf{A}_i \tilde{\mathbf{x}}_i = \mathbf{b}_i$. Therefore, this solution can be written as a sum of the particular solution $\mathbf{x}_i^0$ and a solution of the homogeneous system $\mathbf{A}_i \tilde{\mathbf{x}}_i = 0$. Since $\mathbf{A}_i \mathbf{N}_i = 0$, the column space of $\mathbf{N}_i$ is exactly the null space of $\mathbf{A}_i$. Then $\tilde{\mathbf{x}}_i$ can be written as $\tilde{\mathbf{x}}_i = \mathbf{x}_i^0 + \mathbf{N}_i \tilde{\mathbf{y}}_i$, and it follows that $\mathbf{G}'_i \tilde{\mathbf{y}}_i = \mathbf{G}_i \mathbf{N}_i \mathbf{R}_i \tilde{\mathbf{y}}_i + \mathbf{G}_i \mathbf{x}_i^0 - \mathbf{G}_i \mathbf{x}_i^0 = \mathbf{G}_i \tilde{\mathbf{x}}_i - \mathbf{G}_i \mathbf{x}_i^0 \leq_{\mathcal{K}_i} \mathbf{h}_i - \mathbf{G}_i \mathbf{x}_i^0 = \mathbf{h}'_i$. Therefore, $\tilde{\mathbf{y}}_i$ is a feasible solution of (24) and $s_i^* \leq \frac{1}{2}\tilde{\mathbf{y}}_i^\top \mathbf{H}'_i \tilde{\mathbf{y}}_i + \mathbf{c}_i^\top \tilde{\mathbf{y}}_i + a'_i$. Note

that if $\mathbf{x}_i = \mathbf{N}_i\mathbf{y}_i + \mathbf{x}_i^0$, $\frac{1}{2}\mathbf{y}_i^\top \mathbf{H}_i'\mathbf{y}_i + \mathbf{c}_i'^\top \mathbf{y}_i + a_i' = \frac{1}{2}\mathbf{x}_i^\top \mathbf{H}_i\mathbf{x}_i + \mathbf{c}_i^\top \mathbf{x}_i + a_i$ always holds considering the definition of $\mathbf{H}_i'$, $\mathbf{c}_i'$ and $a_i'$. We can conclude that $s_i^* = \frac{1}{2}\mathbf{x}_i^{*\top}\mathbf{H}_i\mathbf{x}_i^* + \mathbf{c}_i^\top \mathbf{x}_i^* + a_i \le \frac{1}{2}\tilde{\mathbf{x}}_i^\top \mathbf{H}_i\tilde{\mathbf{x}}_i + \mathbf{c}_i^\top \tilde{\mathbf{x}}_i + a_i$. Therefore, $\mathbf{x}_i^*$ is the optimizer of (23), at which the objective takes the optimum $s_i^*$. □

## Supplementary Note 3: Description of the test case

The real-world 10kV distribution network is located in the economic and technological development area in Bayuquan district, Yingkou City, Liaoning Province, China. We model it as a 60-bus grid comprising 1 transformer substation, 48 terminal industrial customers, and 11 intermediate buses with no loads.

The detailed topology and DES configuration information can be found here in Data.xlsx. Due to confidentiality requirements from the State Grid Liaoning company and the end users, all of the data used in this study have been shifted and added with noises to avoid leakage of private information.

# Supplementary Note 4: Decentralized management results on the real test system

See Supplementary Fig. 2 and Supplementary Fig. 3.

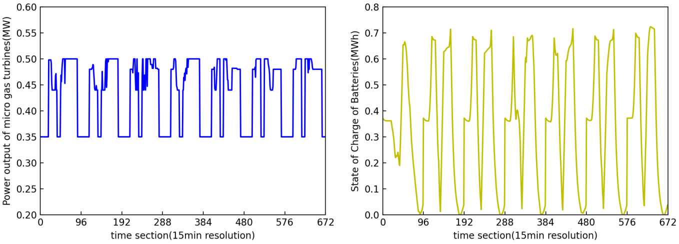


**Supplementary Fig. 2** The power output of micro gas turbines and SOC of batteries owned by local prosumers in the non-cooperative case. Source data are provided as a Source Data file.

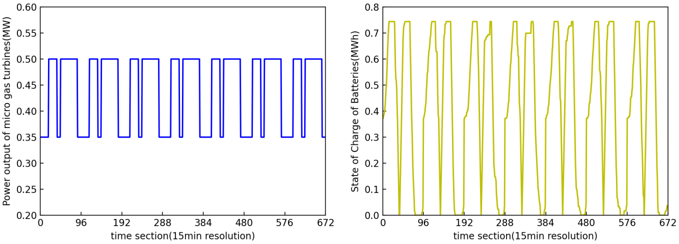


**Supplementary Fig. 3** The power output of micro gas turbines and SOC of batteries owned by local prosumers under decentralized management. Source data are provided as a Source Data file.

# Supplementary Note 5: The test case used in the real communication network and the optimization results.

In the paper we set up a real decentralization platform for illustration of the proposed framework. The test is performed on a test system with 10 DESs. Different from the previous system in Yingkou City, this system involves heat generation and power-heat conversion and storage. Besides, the DESs can operate in self-sufficiency modes. The topology of the system is shown in Supplementary Fig. 4. Supplementary Table 1-3 reveal the configuration of all DESs.

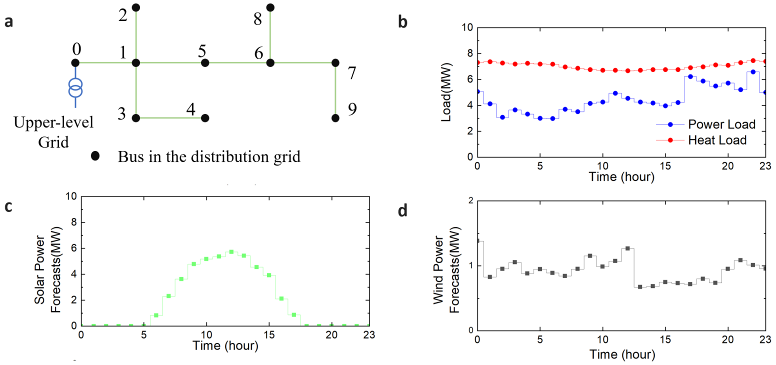


**Supplementary Fig. 4** The 10-bus test case for decentralized management. **a**. The topology of the power distribution grid. The reference bus with number 0 serves as the connection point to the upper-level grid, which establishes fixed buying and selling prices for power exchange with the distribution grid. All of the buses are treated as independent DESs. **b**. The total load of all DESs. **c**. Solar power forecasts of all PV panels. **d**. Wind power forecasts of all wind turbines. Source data are provided as a Source Data file.

**Supplementary Table 1** Configuration of renewable energy sources

| DES | Type | Capacity(MW) | Cost Function | $\tan\phi$ | Capacity Factors |
|---|---|---|---|---|---|
| 7 | Wind Turbine | 2 | $10x^2 + 20x$ | 0.33 | 0.47 |
| 2 | PV panels | 2 | $10x^2 + 20x$ | 0.33 | 0.23 |
| 4 | PV panels | 2 | $10x^2 + 20x$ | 0.33 | 0.23 |
| 6 | PV panels | 2 | $10x^2 + 20x$ | 0.33 | 0.23 |
| 9 | PV panels | 2 | $10x^2 + 20x$ | 0.33 | 0.23 |

All of the line impedances take the nominal value $0.001 + j0.001$pu. The upper-level grid connected at node 0 sell and buy electricity at a constant price of 550 CNY/MWh and 200 CNY/MWh respectively. The convergence curves and the optimization results are shown in Supplementary Fig. 5.

**Supplementary Table 2** Configuration of conventional energy conversion devices

| DES | Type | Capacity(MW) | Cost Function | $\tan\phi$ | Heat-to-power Ratio/COP |
|---|---|---|---|---|---|
| 1 | MGT | 0.5(0.1) | $150x^2 + 400x$ | 0.5 | 0 |
| 2 | MGT | 1.25(0.25) | $150x^2 + 400x$ | 0.2 | 2 |
| 3 | MGT | 0.8(0.2) | $150x^2 + 400x$ | 0.5 | 0 |
| 4 | MGT | 1.25(0.25) | $200x^2 + 370x$ | 0.2 | 2 |
| 4 | HP | 0.5 | / | / | 2 |
| 6 | HP | 1.5 | / | / | 2 |
| 7 | MGT | 1.5(0.3) | $100x^2 + 400x$ | 0.2 | 1 |
| 7 | HP | 1.2 | / | / | 1.8 |

[1]The terms in the brackets represent the lower bound of power generation of the MGTs.

**Supplementary Table 3** Configuration of storage devices

| DES | Type | Capacity(MWh) | Charging/Discharging Power Limit(MW) | $\eta_s$ | $\eta_r$ | $\text{loss}_i(h^{-1})$ |
|---|---|---|---|---|---|---|
| 1 | BATT | 0.2 | 0.1 | 0.9 | 0.9 | / |
| 2 | BATT | 0.2 | 0.1 | 0.9 | 0.9 | / |
| 2 | HS | 1.2 | 0.3 | / | / | 0.01 |
| 3 | BATT | 0.2 | 0.1 | 0.9 | 0.9 | / |
| 8 | BATT | 2 | 0.5 | 0.9 | 0.9 | / |

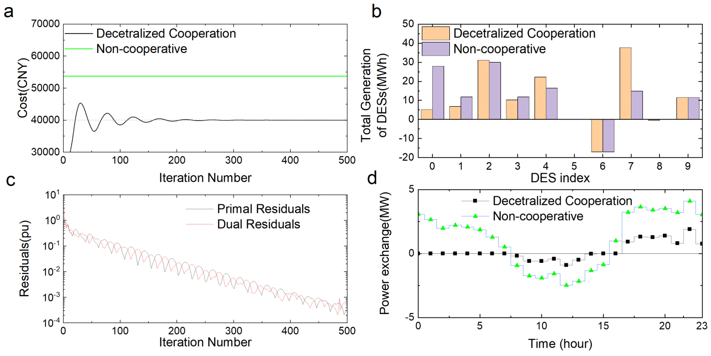


**Supplementary Fig. 5** Optimization results. **a**. The iterative total operating cost of all DESs and the cost in the non-cooperative case. **b**. The primal and dual residuals during the iteration. **c**. Total electricity generation of the 10 DESs over the entire day. **d**. Power exchange of the 10 DESs with the upper-level grid. Source data are provided as a Source Data file.

# Supplementary Note 6: On the behaviour of unfair strategic bidding caused by privacy leakage

In existing blockchain-only schemes, a dishonest miner may colludes with a DES owner and tell him the bidding details of other participants. In this case, the DES owner can simulate the dispatch process and exaggerate its bid parameters for more profits.

For example, in the above 10 DES case, suppose the owner of DES 3 can easily access all details about the other DESs. Through thorough analysis, he can bid by multiplying his true cost function with a strategic bidding parameter. As revealed in Supplementary Fig. 6a, if DES 3 makes a bid 15% higher than its actual cost function, it can reduce the total cost from 2695.23 to 2548.27 CNY, which is about 5.45% lower. This results from the imperfect competition. Since DES 3 is a powerful and efficient participant, claiming a higher cost function will not lead to losing much market share. However, the final marginal cost for settlement will be higher and DES 3 can benefit from such unfair strategic behaviour at the expense of market efficiency. Consequently, the total energy cost of the system increases from 40037.12 to 41268.42 CNY. Some energy consumers have to endure higher costs.

It should be noted that, without privacy leakage, exacerbating bid cost functions can just be treated as a normal market behaviour. DES 3 may either earn more or less. However, privacy leakage makes such strategic bidding very easy and definitely profitable. This is unfair and should be avoided.

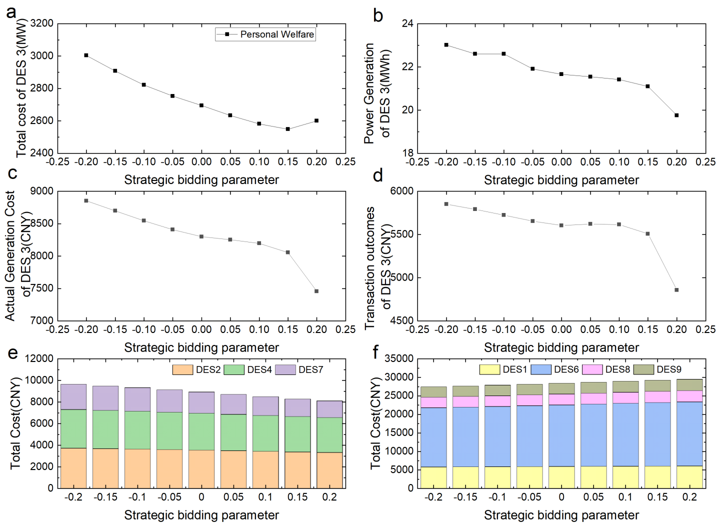


**Supplementary Fig. 6** The impact of strategic bidding of DES 3 on the system. x-axis: the strategic bidding parameter, defined as the ratio of the parameters of the bidding cost function and those of the actual cost function minus 1. **a**. The total running cost of DES 3. **b**. Total power generation of DES 3. **c**. Actual generation cost of DES 3. **d**. DES 3's revenue of selling energy. **e**. Total cost of DES 2, 4, and 7, which benefits from DES 3's exaggerated bidding. **f**. Total cost of DES 1, 6, 8 and 9, which pays more as the bidding parameter increases. DES 0 and DES 5 are not influenced in the test case and are thus not presented. Source data are provided as a Source Data file.

.

# Supplementary Note 7: Discussion about the settlement stage

In this section, we prove that the settlement ensures individual rationality, balanced budget and efficiency.

*Proof* After the convergence of the decentralized iterations, the decision variables of DES i, i.e. $\mathbf{x}_i$ minimizes the objective $\left(\frac{1}{2}\mathbf{x}_{d,i}^\top \mathbf{H}_{d,i}\mathbf{x}_{d,i} + \mathbf{c}_{d,i}^\top \mathbf{x}_{d,i}\right) + \sum_{j\in\mathcal{N}_i}\left[(\boldsymbol{\lambda}_{i,j}^\top(\mathbf{x}_{c,i,j} - \mathbf{e}_{c,i,j}) + \frac{1}{2}\|\mathbf{x}_{c,i,j} - \mathbf{e}_{c,i,j}\|_{\boldsymbol{\Theta}}^2\right]$. Since $\mathbf{x}_{c,i,j} = \mathbf{e}_{c,i,j}$ also holds, $\mathbf{x}_{c,i,j}$ also minimizes $\left(\frac{1}{2}\mathbf{x}_{d,i}^\top \mathbf{H}_{d,i}\mathbf{x}_{d,i} + \mathbf{c}_{d,i}^\top \mathbf{x}_{d,i}\right) + \sum_{j\in\mathcal{N}_i}\boldsymbol{\lambda}_{i,j}^\top \mathbf{x}_{c,i,j}$ if $\boldsymbol{\lambda}_{i,j}$ takes the converged value.

(Individual Rationality) The decentralized scheme offers participants with generation from alternative sources at more affordable prices. Mathematically, the feasible region of decision variable $\mathbf{x}_i$ is enlarged. Therefore, if all of the decision variables and shadow prices are correctly calculated, the final settlement would be profitable for the participants.

(Balanced Budget) For every couple of neighbouring DES $i$ and $j$, $i$ should pay $\boldsymbol{\lambda}_{i,j}^\top \mathbf{x}_{c,i,j}$ to $j$. From the perspective of $j$, its transactive related to $i$ is $\boldsymbol{\lambda}_{j,i}^\top \mathbf{x}_{c,j,i}$. Since $\mathbf{x}_{c,j,i} = \mathbf{x}_{c,i,j}$ and $\boldsymbol{\lambda}_{i,j} + \boldsymbol{\lambda}_{j,i} = 0$ after convergence, we obtain $\boldsymbol{\lambda}_{i,j}^\top \mathbf{x}_{c,i,j} + \boldsymbol{\lambda}_{j,i}^\top \mathbf{x}_{c,j,i} = 0$, which implies balanced budget.

(Efficiency) Efficiency is guaranteed by the convergence of ADMM [5]. After convergence, the selection of $\mathbf{x}_i$ minimizes the overall cost-minimization problem (19).

Incentive compatibility cannot be guaranteed in general. However, if the system of multiple DESs is fully competitive, $\boldsymbol{\lambda}_{i,j}$ will not be affected by the bid of DES $i$. If the price taker $i$ tries to bid strategically, its converged decision variable $\tilde{\mathbf{x}}_i$ would deviate from $\mathbf{x}_i$ which minimizes the cost $\left(\frac{1}{2}\mathbf{x}_{d,i}^\top \mathbf{H}_{d,i}\mathbf{x}_{d,i} + \mathbf{c}_{d,i}^\top \mathbf{x}_{d,i}\right) + \sum_{j\in\mathcal{N}_i}\boldsymbol{\lambda}_{i,j}^\top \mathbf{x}_{c,i,j}$. In this case, the DES participants would prefer to bid truthfully. □

## Supplementary Note 8: Operation algorithm of the DESs and their edge devices

See Supplementary Algorithm 1 for the function of DES $i$'s edge device in iteration $k$.

**Supplementary Algorithm 1** Operation of DES $i$ in iteration $k$

1: **if** $k = 0$ **then**
2: Set $\mathbf{e}_{c,i,j}^{(k-1)}$ and $\boldsymbol{\lambda}_{i,j}^{(k-1)}$ to 0 vector.
3: Calculate the parameters of the masked problem (24) according to (25).
4: Broadcast the parameters $\mathbf{H}'_i$, $\mathbf{c}_i'^{(k)}$, $\mathbf{G}'_i$ and $\mathbf{h}'_i$ to the blockchain miners.
5: **else**
6: Update $\mathbf{c}_i'^{(k)}$ using $\mathbf{e}_{c,i,j}^{(k-1)}$, $\boldsymbol{\lambda}_{i,j}^{(k-1)}$, Eqs. (22), (23) and (25b)
7: Broadcast the parameters $\mathbf{c}'_i$ to the blockchain miners.
8: **end if**
9: Wait until more than 1/3 miners have returned consistent solutions $\mathbf{y}_i^{(k)}$ of (24) of all DESs.
10: Calculate $\mathbf{e}_{c,i,j}^{(k)} := \frac{1}{2}\left(\mathbf{C}_{ij}\mathbf{N}_i\mathbf{R}_i\mathbf{y}_i^{(k)} + \mathbf{C}_{ij}\mathbf{x}_i^0 - \mathbf{C}_{ji}\mathbf{N}_j\mathbf{R}_j\mathbf{y}_j^{(k)} - \mathbf{C}_{ji}\mathbf{x}_j^0\right)$
11: Calculate $\boldsymbol{\lambda}_{i,j}^{(k)} := \boldsymbol{\lambda}_{i,j}^{(k-1)} + \boldsymbol{\Theta}(\mathbf{x}_{c,i,j}^{(k)} - \mathbf{e}_{c,i,j}^{(k)})$

A superscript $(k)$ is used to label terms that depends on the iteration number $k$. Besides, the requirement of more than 1/3 miners originates from the pBFT consensus protocol used in the blockchain.

Intuitively, the DESs are data providers and the edge devices are tamper-proof data preprocessors. They employ the blockchain and the computation workers to solve the encrypted local optimization subproblems in step 9, recover the decision of neighbours in step 10, penalize the inconsistency in cost function in step 11, and update the subproblems in next iterations in step 6.

# Supplementary Note 9: The algorithms executed by miners

See Supplementary Algorithm 2 for the communication module.

---

**Supplementary Algorithm 2** Miner-DES and Miner-worker communication module

---

1: Listen to and record the broadcast from DES $i$: $\mathbf{H}'_i$, $\mathbf{c}'^{(k)}_i$, $\mathbf{G}'_i$ and $\mathbf{h}'_i$.

2: Forward the message to computation workers according to the task allocation schedule.

3: Wait for solutions $\mathbf{y}^{(k)}_i$ from computation workers, forward the message to other miners, and verify their correctness using Karush-Kuhn-Tucker conditions.

4: Record the solution details to the log.

---

Besides, a miner is selected as the leader, who maintains the blockchain using Supplementary Algorithm 3.

The other miners serve as followers, or replicas. They perform Supplementary Algorithm 4, where they can supervise the behaviour of the leader and re-elect the leader in case of fault.

The data structure for each block is shown in Supplementary Fig. 7.

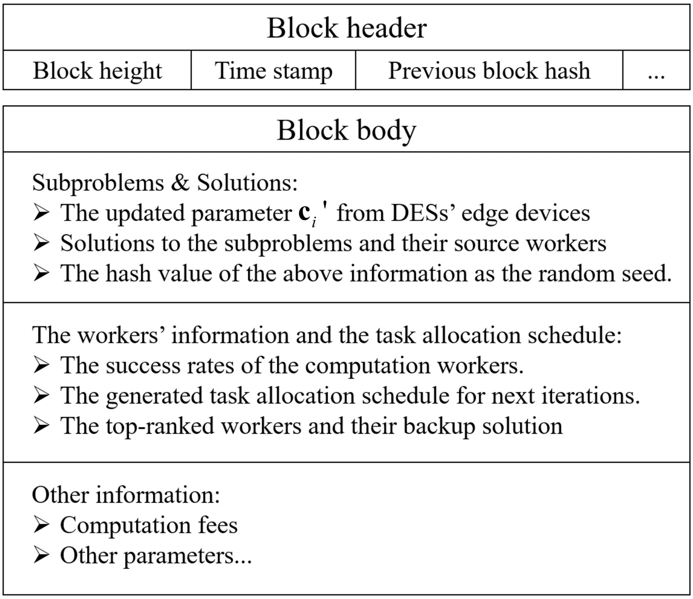


**Supplementary Fig. 7** The data structure of the blocks.

**Supplementary Algorithm 3** Leading miner's blockchain maintenance module

Initialization: A timer $\tau_{\text{ite}}$ to ensure liveness of the iterations, leading miner's index $v$, iteration number $k$.
**while** not converge **do**
  **if** subproblems of all DESs in iteration $k$ have been recorded to log or timeout $t_{ite}$ has expired **then**
    Package all $\mathbf{y}_i^{(k)}$ into a new candidate block. If some $\mathbf{y}_i^{(k)}$ have not been returned by workers, package the latest legal $\mathbf{y}_i^{(k')}$ in previous iterations $(k')$. Update the rates of successful solution (rate$_i$, $0 < \text{rate}_i < 1$) of all computation parties.
    Calculate the hash value of solution results and set it as the random seed. Use the random seed to rearrange the computation tasks and generate a task allocation schedule using the success rates as weights. Let the top-ranked computation parties serve as redundant backup solvers for the subproblems.
    Broadcast the block candidate to other peer miners, initiate the pBFT multi-phase protocol(pre-prepare, prepare, commit, execute) to reach a consensus, and attach the block to the chain.
    **if** the execution is caused by timeout **then**
      multiply $t_{\text{ite}}$ by 2.
    **end if**
    Broadcast the block to DESs.
    Reset the timer and prepare for the next block.
  **end if**
**end while**

**Supplementary Algorithm 4** Following miner's blockchain maintenance module

Initialization: A communication timer $\tau_{lead}$ to ensure liveness of the leading miner, leading miner's index $v$, iteration number $k$.
**while** True **do**
  Listen to the candidate blocks from the leading miner, verify their legality, and follow the pBFT multi-phase protocol to attach it to the chain.
  Whenever the communication with the leading miner times out or the leader makes a mistake, initiate the view-change protocol to re-elect the leading miner.
**end while**

# References


1. Chen, S. *et al.* A blockchain consensus mechanism that uses proof of solution to optimize energy dispatch and trading. *Nature Energy* **7** (6), 495–502 (2022) .

2. Yan, M., Teng, F., Gan, W., Yao, W. & Wen, J. Blockchain for secure decentralized energy management of multi-energy system using state machine replication. *Applied Energy* **337**, 120863 (2023). https://doi.org/https://doi.org/10.1016/j.apenergy.2023.120863 .

3. Baran, M. E. & Wu, F. F. Optimal capacitor placement on radial distribution systems. *IEEE Transactions on Power Delivery* **4** (1), 725–734 (1989). https://doi.org/10.1109/61.19265 .

4. Farivar, M. & Low, S. H. Branch flow model: Relaxations and convexification—part i. *IEEE Transactions on Power Systems* **28** (3), 2554–2564 (2013). https://doi.org/10.1109/TPWRS.2013.2255317 .

5. Boyd, S., Parikh, N., Chu, E., Peleato, B. & Eckstein, J. *Distributed Optimization and Statistical Learning via the Alternating Direction Method of Multipliers* (Now Publishers Inc., 2011).

6. Tang, C., Liu, M., Liu, Q. & Dong, P. A per-node granularity decentralized optimal power flow for radial distribution networks with pv and ev integration. *International Journal of Electrical Power and Energy Systems* **116** (2020). https://doi.org/10.1016/j.ijepes.2019.105513 .

7. Sun, Q., Zhao, T., Chen, Q., He, K. & Ma, H. Decentralized dispatch of distributed multi-energy systems with comprehensive regulation of heat transport in district heating networks. *IEEE Transactions on Sustainable Energy* **14** (1), 97–110 (2022) .
