# Supplementary material for: Break down the decentralization-security-privacy trilemma in management of distributed energy systems
Source: Nat Commun. 2024 May 27;15:4508. doi: 10.1038/s41467-024-48860-7 (PMC11130155; doi:10.1038/s41467-024-48860-7)
Supplement: Supplementary file 3 — Reporting Summary [file 41467_2024_48860_MOESM3_ESM.pdf]

nature portfolio

Corresponding author(s): Qun Chen

Last updated by author(s): Mar 3, 2024

Reporting Summary

Nature Portfolio wishes to improve the reproducibility of the work that we publish. This form provides structure for consistency and transparency in reporting. For further information on Nature Portfolio policies, see our Editorial Policies and the Editorial Policy Checklist.

Statistics

For all statistical analyses, confirm that the following items are present in the figure legend, table legend, main text, or Methods section.

n/a	Confirmed	
☐	☒	The exact sample size (*n*) for each experimental group/condition, given as a discrete number and unit of measurement
☐	☒	A statement on whether measurements were taken from distinct samples or whether the same sample was measured repeatedly
☒	☐	The statistical test(s) used AND whether they are one- or two-sided<br>*Only common tests should be described solely by name; describe more complex techniques in the Methods section.*
☒	☐	A description of all covariates tested
☒	☐	A description of any assumptions or corrections, such as tests of normality and adjustment for multiple comparisons
☒	☐	A full description of the statistical parameters including central tendency (e.g. means) or other basic estimates (e.g. regression coefficient) AND variation (e.g. standard deviation) or associated estimates of uncertainty (e.g. confidence intervals)
☒	☐	For null hypothesis testing, the test statistic (e.g. F, t, r) with confidence intervals, effect sizes, degrees of freedom and P value noted<br>*Give P values as exact values whenever suitable.*
☒	☐	For Bayesian analysis, information on the choice of priors and Markov chain Monte Carlo settings
☒	☐	For hierarchical and complex designs, identification of the appropriate level for tests and full reporting of outcomes
☒	☐	Estimates of effect sizes (e.g. Cohen's *d*, Pearson's *r*), indicating how they were calculated

Our web collection on statistics for biologists contains articles on many of the points above.

Software and code

Policy information about availability of computer code

Data collection	The raw data used for this study was recorded by the smart meters owned by State Grid Liaoning Electric Power Supply Co., Ltd and stored by State Grid Liaoning Electric Power Research Institute. We copied the data from their database. No software was used.
Data analysis	The code used for this study, as well as descriptions of the dependent libraries and versions can be found at https://cloud.tsinghua.edu.cn/d/4217a67c8f32465cb231/ .

For manuscripts utilizing custom algorithms or software that are central to the research but not yet described in published literature, software must be made available to editors and reviewers. We strongly encourage code deposition in a community repository (e.g. GitHub). See the Nature Portfolio guidelines for submitting code & software for further information.

Data

Policy information about availability of data

All manuscripts must include a data availability statement. This statement should provide the following information, where applicable:

- Accession codes, unique identifiers, or web links for publicly available datasets
- A description of any restrictions on data availability
- For clinical datasets or third party data, please ensure that the statement adheres to our policy

The data used for this study are all available at https://cloud.tsinghua.edu.cn/d/4217a67c8f32465cb231/ .

Research involving human participants, their data, or biological material

Policy information about studies with human participants or human data. See also policy information about sex, gender (identity/presentation), and sexual orientation and race, ethnicity and racism.

Reporting on sex and gender	This is not a research involving human participants, and this item is not relevant to our study.
Reporting on race, ethnicity, or other socially relevant groupings	This is not a research involving human participants, and this item is not relevant to our study.
Population characteristics	This is not a research involving human participants, and this item is not relevant to our study.
Recruitment	This is not a research involving human participants, and this item is not relevant to our study.
Ethics oversight	This is not a research involving human participants, and this item is not relevant to our study.

Note that full information on the approval of the study protocol must also be provided in the manuscript.

Field-specific reporting

Please select the one below that is the best fit for your research. If you are not sure, read the appropriate sections before making your selection.

☐ Life sciences ☒ Behavioural & social sciences ☐ Ecological, evolutionary & environmental sciences

For a reference copy of the document with all sections, see nature.com/documents/nr-reporting-summary-flat.pdf

Behavioural & social sciences study design

All studies must disclose on these points even when the disclosure is negative.

Study description	We propose a new decentralized energy management method to optimize multiple distributed energy systems and use the real data of a power distribution grid in Liaoning Province, China to perform the case study and test the effectiveness of the proposed method. Our work is not a behavioural or social sciences study, but we still describe the test case in below:<br>The grid topology, raw load, renewable power output and local generation information are quantitative data provided by State Grid Liaoning Electric Power Supply Co., Ltd and State Grid Liaoning Electric Power Research Institute.
Research sample	The research sample is a 60-bus distribution grid in the economic devlopement area of Yingkou City, Liaoning Province. The distribution grid involves many industrial and commercial energy producers and consumers. Therefore, we use its historic operation data to test the proposed method.
Sampling strategy	We randomly select one week data from 6 Nov 2023 to 12 Nov 2023 to perform the study.
Data collection	The raw data is recorded by the user-side smart meters owned by State Grid Liaoning Electric Power Supply Co., Ltd. and stored by State Grid Liaoning Electric Power Research Institute. We collected the data from their database.
Timing	6 Nov 2023 to 12 Nov 2023, with a time resolution of 15min.
Data exclusions	No data was excluded
Non-participation	This item is not applicable to our research.
Randomization	This item is not applicable to our research. There are no participants and we involve no allocation.

Reporting for specific materials, systems and methods

We require information from authors about some types of materials, experimental systems and methods used in many studies. Here, indicate whether each material, system or method listed is relevant to your study. If you are not sure if a list item applies to your research, read the appropriate section before selecting a response.

Materials & experimental systems

n/a	Involved in the study
☒	☐ Antibodies
☒	☐ Eukaryotic cell lines
☒	☐ Palaeontology and archaeology
☒	☐ Animals and other organisms
☒	☐ Clinical data
☒	☐ Dual use research of concern
☒	☐ Plants

Methods

n/a	Involved in the study
☒	☐ ChIP-seq
☒	☐ Flow cytometry
☒	☐ MRI-based neuroimaging

Plants

Seed stocks

This is not a research involving plants, and this item is not relevant to our study.

Novel plant genotypes

This is not a research involving plants, and this item is not relevant to our study.

Authentication

This is not a research involving plants, and this item is not relevant to our study.
